# A path-based measurement for human miRNA functional similarities using miRNA-disease associations

**DOI:** 10.1038/srep32533

**Published:** 2016-09-02

**Authors:** Pingjian Ding, Jiawei Luo, Qiu Xiao, Xiangtao Chen

**Affiliations:** 1 College of Computer Science and Electronic Engineering, Hunan University, Changsha, 410083, China

## Abstract

Compared with the sequence and expression similarity, miRNA functional similarity is so important for biology researches and many applications such as miRNA clustering, miRNA function prediction, miRNA synergism identification and disease miRNA prioritization. However, the existing methods always utilized the predicted miRNA target which has high false positive and false negative to calculate the miRNA functional similarity. Meanwhile, it is difficult to achieve high reliability of miRNA functional similarity with miRNA-disease associations. Therefore, it is increasingly needed to improve the measurement of miRNA functional similarity. In this study, we develop a novel path-based calculation method of miRNA functional similarity based on miRNA-disease associations, called MFSP. Compared with other methods, our method obtains higher average functional similarity of intra-family and intra-cluster selected groups. Meanwhile, the lower average functional similarity of inter-family and inter-cluster miRNA pair is obtained. In addition, the smaller p-value is achieved, while applying Wilcoxon rank-sum test and Kruskal-Wallis test to different miRNA groups. The relationship between miRNA functional similarity and other information sources is exhibited. Furthermore, the constructed miRNA functional network based on MFSP is a scale-free and small-world network. Moreover, the higher AUC for miRNA-disease prediction indicates the ability of MFSP uncovering miRNA functional similarity.

In recent years, more and more researchers pay attention to measure miRNA functional similarity in biology research, since it is very important for many applications such as miRNA clustering^1^, miRNA function prediction^2^, miRNA synergism identification^3^,^4^,^5^,^6^, miRNA-mRNA interaction inference^7^ and disease miRNA prioritization^8^,^9^,^10^,^11^,^12^,^13^. Although miRNA similarity could be calculated based on miRNA sequence or expression data^6^,^14^, functional similarity is more beneficial to fully understand the functions and biological roles of miRNA^15^.

In previous studies, some computational approaches based on miRNA-target associations were developed for studying miRNA functional similarity. Shalgi *et al*.^16^ utilized Jaccard similarity method to quantify the functional similarity of two miRNAs based on their common target genes. However, most of miRNA functional similarities using the Jaccard similarity measurement are zeros, because there is no intersection among the target gene sets of most human miRNAs^17^,^18^. Yu *et al*.^19^ systematically measured the functional similarity of miRNA pair using GO annotation of their target genes. Sun *et al*.^20^ calculated the miRNA functional similarity based on miRNAs targeting propensity and proteins connectivity in the integrated protein-protein interaction network. Xu *et al*.^21^ combined the site accessibility and the interactive context of target genes in functional gene networks, which was constructed with semantic similarity weights generated from the GO terms of the target genes, to infer the functional similarity of miRNA pair. Meng *et al*.^22^ proposed a method, called PPImiRFS, for calculating the functional similarity of plant miRNAs inferred from similarity of their target gene sets. PPImiRFS firstly constructed a protein-protein interaction network using the gene semantic similarity by GOSemSim^23^ and then quantified the functional similarity of target pair based on the shortest paths. A modified best-match average method was employed to calculate the functional similarity of miRNA pair using the predicted miRNA gene^24^. In addition, Xu *et al*.^3^ defined miRNA synergistic pair which significantly related to at least one co-regulating functional module established by a protein-protein interaction network^25^,^26^. Nevertheless, this method could not measure the level of miRNA similarity, since the calculated similarities are only 0 or 1. Moreover, the above methods always make use of the predicted miRNA target which has high false positives and false negatives. It makes these methods difficult to obtain high reliability for miRNA functional similarity. So, Liu *et al*.^27^ integrated the miRNA-target association, the lncRNA-disease association and the miRNA-lncRNA association to quantify the miRNA similarity. However, there is informative subtype information of disease verified by expertise in the US National Library of Medicine^28^. Furthermore, lncRNA-disease associations are always utilized to calculate lncRNA functional similarity in many methods^29^,^30^,^31^ and the density of miRNA-disease associations validated by biology experiment is greater than that of miRNA target in human. Therefore, Wang *et al*.^32^ utilized human miRNA-disease associations to compute the functional similarity scores based on the supposition that similar miRNAs tend to be associated with similar diseases, named MISIM. In this method, an inferring GO term similarity algorithm was applied to measure the semantic similarity of diseases structured as directed acyclic^33^, and then the miRNA functional similarity was inferred by best-match average (BMA) method. Moreover, Xuan *et al*.^34^ improved the calculation of information content (IC) of diseases based on the intuition that the more general the disease term is and the less semantic contribution it has, which ensure higher reliability of semantic similarity of disease. Similarly, BMA method was employed to quantify the miRNA functional similarity based on disease similarity. However, these methods using miRNA-disease associations, namely based on BMA method, did’nt consider the topology of disease semantic network. Furthermore, path-based similarity measurements have been successfully applied on various types of relationship data^35^,^36^. Therefore, an efficiently path-based method is required to measure miRNA functional similarity using miRNA-disease associations.

In this study, we designed a method, called MFSP (MiRNA Functional Similarity based on Path), to infer the functional similarity of miRNA pair using miRNA-disease associations. To validate MFSP, we compared it with two other state-of-the-art methods based on disease-related miRNAs, namely Wang’s method^32^ and Xuan’s method^34^, with functional similarity scores of intra-family, inter-family, intra-cluster and inter-cluster miRNA pairs. Meanwhile, the lower p-value was obtained while applying Wilcoxon rank-sum test and Kruskal-Wallis test on different miRNA groups. Furthermore, it was verified that the positive correlation is between the expression similarity and the functional similarity. The negative effect of distance of genome coordinate was exhibited for functional similarity. In addition, the effect of varying parameters was analyzed. For formed miRNA network based on miRNA functional similarity, it is scale-free network and small world network. The higher AUC was achieved while MIDP^9^ was applied to the miRNA network constructed by MFSP. Moreover, a Cosine Similarity of Disease, named CSD, was developed to improve the reliability of dieasese semantic similarity. To validate CSD, the different similarity calculation methods of disease and BMA method for miRNA functional similarity calculation were combined to compare performance.

## Results

### Design of experiment

MFSP was developed to infer miRNA functional similarity by combining subtype information of disease obtained from MeSH (http:www.ncbi.nlm.nih.gov/)^28^ and the known miRNA-disease associations provided by HMDD^37^. In the MFSP method, first, the semantic similarity of disease is calculated based on subtype information of disease. Then, unlike the Wang’s method^32^ and Xuan’s method^34^ which are only consider the direct neighbor diseases of miRNA, MFSP measures the miRNA functional similarity based on the topology of disease network constructed by semantic similarity of disease. Furthermore, functional similarities of all miRNA pairs are provided in Supplementary Material 1. The performance of MFSP was evaluated by average similarity of different miRNA groups and p-value obtained by Wilcoxon rank-sum test and Kruskal-Wallis test. The relationships between other biology informations and miRNA functional similarity were exhibited. The results of predicting disease-related miRNAs were compared. In addition, we analyzed the effect of parameters and constructed miRNA network based on miRNA functional similarity calculated by MFSP.

### Performance

**Figure 1:**
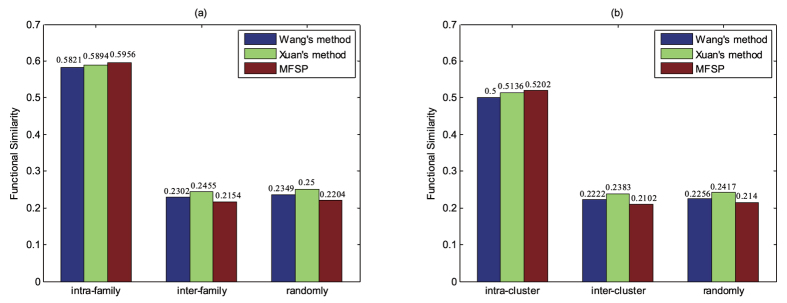
Similarity comparison for MFSP and other methods using miRNA family and miRNA cluster.

The miRNA-disease associations dataset of human used in the experiment can be downloaded from HMDD^37^. The latest version of HMDD (updated in 2015) includes 330 diseases which contained in the US National Library of Medicine (MeSH) and their associated 574 miRNAs (Supplementary Material 2). After a comprehensive exploration, the performances of MFSP and the existing methods are compared intuitively while parameters *a* = 0.6 and *b* = 5. A family of miRNAs exhibit sequence similarity and has completed identical seed regions. Therefore, miRNAs in the same family are likely to show high functional similarity. MiRNAs belonging to the same family are provided by RFam^38^. In order to measure the performance of MFSP, the human miRNAs are divided three classes: intra-family, inter-family and randomly selected miRNA group which included intra-family pairs and inter-family pairs (76 families that contain 534 miRNAs). The computed functional similarity scores are shown in Fig. 1(a). It can be seen that, in the intra-family selected miRNA groups, the functional similarity score calculated by MFSP is higher than that of Wang’s method^32^ and Xuan’s method^34^, which implemented with the same version of database. Meanwhile, the functional similarity score calculated by MFSP is lower than that of other methods in terms of inter-family groups.

In addition, we applied Wilcoxon rank-sum test and Kruskal-Wallis test^22^ implemented by Matlab to demonstrate significant differences (Table 1). As shown in the Table 1, the functional similarity of intra-family group was significantly greater than that of inter-family (Wilcoxon rank-sum test; intra-inter family p-value = 0.00E-00). The result of Kruskal-Wallis test further clarified that our method is effective (p-value = 0.00E-00). Furthermore, the smaller p-value calculated by Wilcoxon rank-sum test between inter-family and randomly miRNA pairs confirmed that our method performed better than other methods.

In addition, to validate the advantage of CSD compared with other methods using disease DAG, we integrated CSD with BMA to infer the miRNA similarity since the difference between Wang’s method and Xuan’s method is disease similarity calculation and BMA was employed in the two methods for miRNA functional similarity. From Table 1, the lower p-value is obtained by CSD+BMA, which demonstrates that CSD is more effective than two other methods on calculating the disease semantic similarity.

Mature miRNAs in the same cluster tend to transcribe and express synchronously. Therefore, miRNAs with the higher functional similarity tend in the same cluster. Similarly, we selected 50 kb for genome coordinate data, which are downloaded from miRBase^39^, as a distance cutoff separated into three classes: intra-cluster, inter-cluster and randomly selected miRNA group which contained intra-cluster and inter-cluster pairs (77 clusters that include 574 miRNAs). The computed functional similarity of intra-cluster, inter-cluster and randomly miRNAs are shown in Fig. 1(b). Compared with other methods, the functional similarity of intra-cluster group obtained by MFSP is higher. The functional similarity of inter-cluster achieved by MFSP is lower than that of other methods simultaneously. Similarly, Wilcoxon rank-sum test and Kruskal-Wallis test were applied for intra-cluster group, inter-cluster group and randomly group to demonstrate significant differences (Table 2). The smaller p-values were obtained by MFSP based on Wilcoxon rank-sum test and Kruskal-Wallis test. It further demonstrated that the performance of MFSP is better than the calculated results of other methods. In addition, CSD+BMA method also could receive lower p-value compared with Wang’s method and Xuan’s method for intra-cluster, inter-cluster and randomly groups, which indicated that CSD may be a better method for disease semantic similarity calculation using DAG of disease.

**Figure 2:**
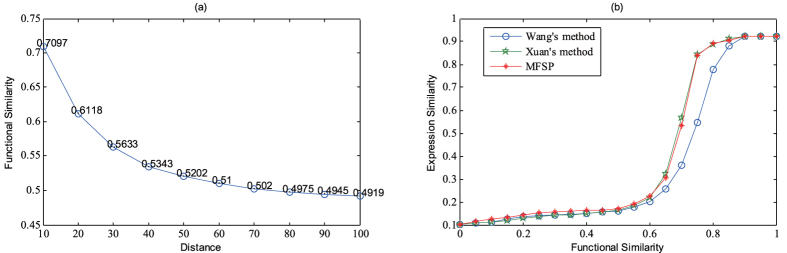
The relationship between miRNA functional similarity and other information sources of miRNA. (**a**) The relationship between distance cutoff for identifying miRNA clusters and miRNA MFSP functional similarity. (**b**) The relationship between expression similarity and miRNA MFSP functional similarity.

MiRNAs with similar functions tend to be located in the nearby genome coordinate. We grouped miRNAs into different clusters using distance cutoffs from 10 kb to 100 kb by a step of 10 kb. Then, the average functional similarity of intra-cluster miRNA pairs were calculated as shown in the Fig. 2(a). It can be seen that the functional similarity calculated by MFSP is negatively correlated with the distance of genome coordinate.

MiRNAs with similar functions are likely to act on the similar cellular components and relate to similar biological processes. Therefore, miRNA with similar functions tend to have similar expression profiles. The expression profiles of 345 miRNAs across 40 normal human tissues were obtained from the supplementary files of the paper by Liang *et al*.^40^. PCC (Pearson Correlation Coefficient) was used as the measurement for expression similarity of miRNAs. MiRNAs were grouped into different groups according the threshold value *t*. It ranges in [0, 1] and the step is 0.05. Then, we calculated the average expression similarity of miRNA pairs whose functional similarity is higher than the threshold value *t*. As shown in Fig. 2(b), the functional similarity calculated by MFSP is positively correlated with expression similarity of miRNA. In addition, Pearson correlation coefficient (PCC) is employed to numerically demonstrate the relationship between the functional similarity and the miRNA expression similarity (R = 0.8792, P = 0.1551E-06 for MFSP; R = 0.866, P = 0.3919E-06 for Wang’s method; R = 0.8835, P = 0.1112E-06 for Xuan’s method). In respect of correlation coefficient, MFSP is similar to Xuan’s method and better than Wang’s method.

### Parameter analysis

**Figure 3:**
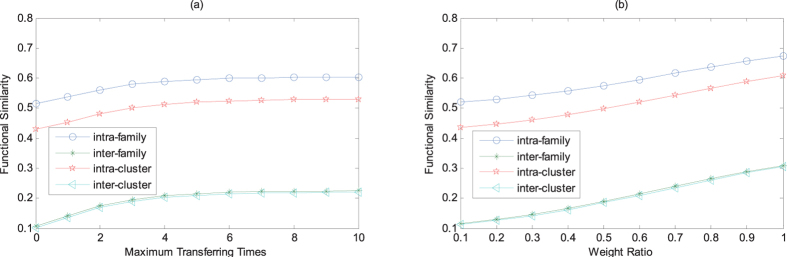
MFSP functional similarity with different maximum transferring times and weight ratio.

Two issues about the maximum transferring times *b* and the weight ratio *a* are analyzed in the experiment for MFSP. MFSP was implemented varying the maximum transferring times *b* while the weight ratio *a* is 0.6. As can be seen in Fig. 3(a), the first few transfer times can effectively boost the similarity of miRNA pairs containing intra-family, inter-family, intra-cluster and inter-cluster pairs. In addition, the similarity of miRNA pair is stabilized as the maximum transferring times increase to 5. In Supplementary Table 1, the p-value keeps decreasing expect b ≤ 1 as the maximum transferring times gradually increase Table 3.

To test the impact of the weight ratio *a*, we vary the value of the weight ratio and set *b* = 5. Results were shown in Fig. 3(b), where it plotted the average similarity of miRNA pairs with different weight ratio. It can be seen that the average similarity of miRNA pairs included intra-family, inter-family, intra-cluster and inter-cluster pairs keeps getting growth as the weight ratio increase. Furthermore, these functional similarity scores for varying weight ratio demonstrate significant differences based on Kruskal-Wallis test and Wilcoxon rank-sum test (Supplementary Table 2).

### Construction of miRNA network

**Figure 4:**
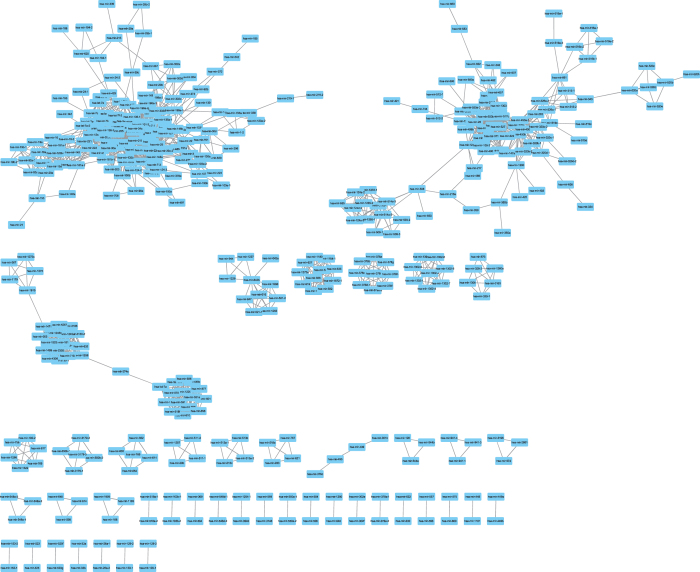
MiRNA functional network constructed by miRNA functional similarity.

We construct the miRNA functional network based on the criterion that an edge is added to link two miRNAs whose functional similarity is greater than 0.7. The threshold is set as 0.7 based on three reasons. Firstly, the average functional similarity of miRNA pairs within 10 kb for genome coordinate is about 0.7. Secondly, while functional similarity of miRNA pair is greater than 0.75, its expression similarity raises slowly. Thirdly, while the threshold is 0.7 rather than 0.75, 85 more miRNAs are contained in the constructed network. The constructed miRNA network includes 422 nodes and 1794 edges and is visualized by Cytoscape (Fig. 4). As shown in Supplementary Figure 1, there are a large number of miRNA partners for a few miRNAs, whereas many miRNAs interact with few miRNA partners. A power law fitting is performed to demonstrate that the node degree follows a power law with a slope of −1.165 and R-squared is 0.676, as expected for a scale-free network (Supplementary Figure 1). Furthermore, the “Random Networks” plugin of Cytoscape^41^ is executed to achieve the topological measurements and random networks. We find that the characteristic path length of 3.358 for the constructed miRNA functional network is similar to that of random graphs generated by the ER model (3.35035 ± 0.0231). Meanwhile, the higher average clustering coefficient of 0.595 is obtained compared the constructed network with random networks (0.1318098 ± 0.0060293), which demonstrate that the miRNA functional network is a small-world network^42^. In addition, the CFinder is implemented^43^ to infer cliques which are all of complete sub-graphs in the miRNA functional network. As shown in Supplementary Figure 2, with an increase in the value of *k*, the number of cliques is on rise peaking at 29598 while the *k* value is 7. However, the number of cliques decreases while the *k* value continues to grow. This phenomenon is according with a principle that the specific regulation is implemented by small clusters rather than individual or big modules.

### Application

In order to verify the effectiveness of MFSP comparing with other methods, MIDP^9^ which walks on the miRNA similarity network predicts miRNA-disease associations on miRNA network constructed by different methods (*r*_*Q*_ = 0.4, *r*_*U*_ = 0.1 for MIDP). We performed experiments using leave-one-out cross validation (LOOCV) scheme which is always used on prediction of miRNA-disease association^13^. In previous studies^8^,^9^,^10^,^11^,^12^,^13^, the area under the ROC^44^ curve (AUC) was employed as the main metric for performance evaluation. Five diseases with more than 160 related miRNAs were performed in the experiment (Melanoma, Hepatocellular Carcinoma, Breast Neoplasms, Colorectal Neoplasms, Stomach Neoplasms), since disease with a few miRNAs was not sufficient to evaluate the prediction performance. As a result, our method achieved the average AUC of 0.95578 which is higher than that of other methods (Table 3). The performance of MFSP for all five diseases is superior to other methods except AUC of hepatocellular carcinoma calculated by Wang’s method. It indicated that our method is preferable for predicting miRNA-disease association based on miRNA similarity network. In addition, using MFSP rather than MISIM to calculate the miRNA functional similarity, results of other prediction methods (such as WBSMDA^45^ and KATZ^46^) may be improved.

## Discussion

In this paper, we presented a path-based calculation method of the miRNA-miRNA functional similarity, called MFSP. It measured the miRNA functional similarity based on the paths among miRNA-related disease sets. The similarities of intra-family, inter-family, intra-cluster and inter-cluster miRNA groups were compared for MFSP and other state-of-the-art methods. The superior effectiveness of MFSP was verified by Wilcoxon rank-sum test and Kruskal-Wallis test. Furthermore, we demonstrated the negative correlation between distance of genome coordinate and miRNA functional similarity and the positive correlation between expression similarity and miRNA functional similarity. The constructed miRNA network based on functional similarity is a scale-free and small-world network. For application of miRNA-disease association prediction, MIDP could obtain higher AUC based on miRNA functional similarity calculated by MFSP. In addition, MFSP could be applied to calculate lncRNA functional similarity using lncRNA-disease associations^29^,^30^,^31^. It also could infer gene functional similarity based on GO terms^33^, which may contribute to the performance of predicting disease-related target^47^.

Moreover, we proposed a calculation for disease semantic similarity, called CSD, which transformed all structural disease data into attribute feature vector of disease and then calculated the semantic similarity by traditional cosine similarity. For experimental results, when CSD is employed rather than other calculation methods, results of BMA could be improved.

As future work, other biological information may contribute to further improve the reliability of miRNA functional similarity. For example, instead of using just miRNA-disease association, multiple associations including miRNA-target association and miRNA-disease association may be used for miRNA functional similarity calculation.

## Methods

### Method Overview

**Figure 5:**
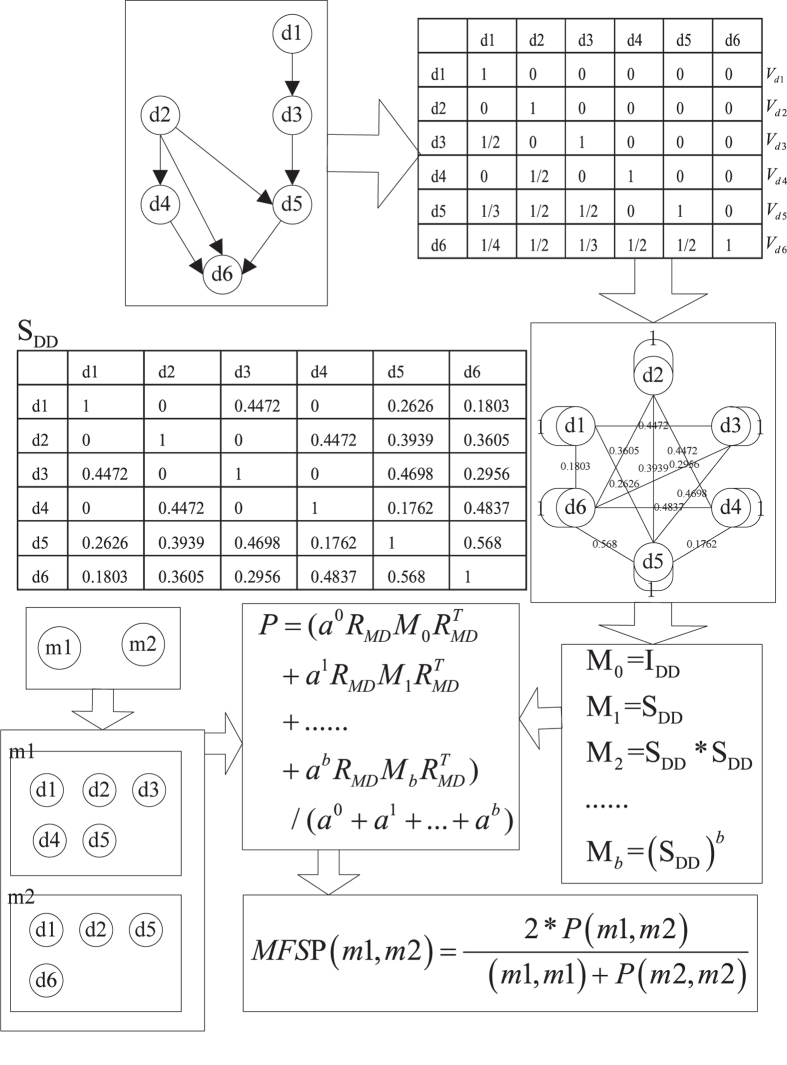
The flow chart of MFSP.

In this study, we presented a method, MFSP, to measure the functional similarity of miRNA pair. The flow chart of MFSP is shown in Fig. 5. First, the hierarchical structure about disease obtained from MeSH descriptor was transferred into features of diseases. Second, the semantic similarity of disease pair was calculated by cosine similarity and the disease similarity network was constructed. Third, the weight sum of paths among diseases was achieved based on the different transferring times respectively. Forth, the miRNA-miRNA path matrix was formed based on the weight sum of pathes among disease sets. Finally, we measured the functional similarity by miRNA-miRNA path matrix. Details of the procedures are given in the following sections.

### Cosine Similarity of Disease (CSD)

**Figure 6:**
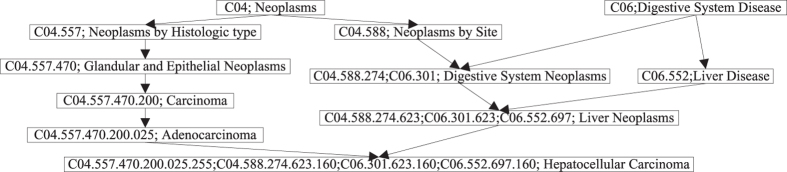
Hierarchical DAG of Hepatocellular Carcinoma.

The procedures of CSD are mainly composed of two steps: 1) attribute feature of disease is obtained; 2) cosine similarity is employed. Hierarchical DAG, in which the nodes represent diseases while the links represent relationship between nodes, is provided by the MeSH database (http:www.ncbi.nlm.nih.gov/). Only one type of relationship which represents a child node connecting to a parent node is contained in the DAG. Each disease corresponds to one or more MeSH ID which numerically defines its location in MeSH graph. The codes of a child node are composed of the codes of its parent nodes and the child’s addresses. For instance as shown in Fig. 6, MeSH IDs: C06.301 and C04.588.274 are all corresponding to Digestive System Neoplasms. The entries on its parent nodes (Digestive System Diseases and Neoplasms by Site) have only MeSH ID: C06 and C04.588, respectively.

In order to apply the traditional methods (similarity measurement, classification and link prediction, and so on) in relational database, the relational structure are always transferred into attribute features of object^48^,^49^. In this study, we represent a disease *d* as a vector *V*_*d*_ of size 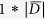, where 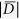 is the number of diseases in the MeSH database. The *i*-th element of *V*_*d*_ is defined as:


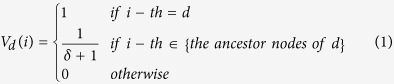


where *δ* is the shortest distance between *i*-th node, an ancestor node of *d*, and the disease *d*. Therefore, the shorter distance of the ancestor node from node *d* is, namely the more specific denomination of the ancestor node is, the higher value corresponding to the ancestor node will be. For example as shown in Fig. 6, Neoplasms (C04) is an ancestor node of Hepatocellular Carcinoma (C04.588.274.623.160, C04.557.470.200.025.255). The length of two paths from Neoplasms to Hepatocellular Carcinoma is 4 and 5, respectively. Therefore, the element of feature vector for Hepatocellular Carcinoma corresponding to Neoplasms is 1/(4 + 1) = 1/5. Based on the feature vector of each disease formed by equation (1), we then calculated the semantic similarity of disease pair *d*_*i*_ − *d*_*j*_ by cosine similarity:


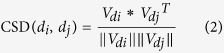


where *V*_*di*_ and *V*_*dj*_ are the feature vector of disease *d*_*i*_ and *d*_*j*_, respectively. The disease similarity network can be constructed via the semantic similarity of disease pair.

### MiRNA functional similarity

Many path-based similarity measurements are utilized in the relationship data and verified effectively^35^,^36^. To obtain the more accurate similarity of miRNA pair using paths between disease sets, we need to consider the topological structure of disease network and diseases related to these two miRNAs. Therefore, human miRNA-disease associations are downloaded from HMDD database updated in 2015^37^, which contained 6197 distinct human miRNA-disease associations among 330 diseases, which are included in MeSH, and their related 574 miRNAs. Assumed that |*M*| and |*D*| denote the number of miRNAs and diseases in the HMDD respectively, matrix *R*_*MD*_ of size 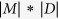 represents the adjacency matrix of miRNA-disease association, where the entry *R*_*MD*_(*i*, *j*) in the row *i* column *j* is 1 if miRNA *i* is related to disease *j*, 0 otherwise. Matrix *S*_*DD*_ of size 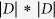 is the similarity matrix of disease and its element *S*_*DD*_(*d*_*i*_, *d*_*j*_) represents the semantic similarity *CSD*(*d*_*i*_, *d*_*j*_) of *d*_*i*_ − *d*_*j*_ pair. In this study, we defined transferring matrix to describe the topological structure of the disease similarity network. Given the maximum transferring times *b*, transferring matrix *M*_*i*_ for each transferring times *i* is defined as follows:


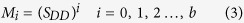


Assume that the weight of path is the product of weights of links on it, transferring matrix *M*_*i*_ (*i* denotes transferring times) is a symmetric matrix, whose each element representing the sum of the *i*-length path weight for the disease pair.

Here, we utilized the miRNA-miRNA path matrix *P* to describe the sum of weights of paths between disease sets related to miRNA pair. Since longer path utilized more remote relationships, it was assigned a smaller weight. The different transferring times on the disease similarity networks are combined with a weight ratio *a* to dampen the contributions from longer paths (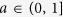). Consequently, the miRNA-miRNA path matrix *P* is defined as follows:


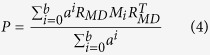


Based on the formula (4), we can see that a longer path namely larger transferring times may contribute less than a shorter path namely smaller transferring times. The element *P*(*m*_*i*_, *m*_*j*_) denotes the sum of the weight of the *b* maximum-length paths from *m*_*i*_-related diseases to *m*_*j*_-related diseases. Then, MFSP between miRNA *m*_*i*_ and *m*_*j*_ can be calculated as:


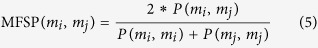


The formula (5) shows that *MFSP*(*m*_*i*_, *m*_*j*_) is composed of two parts: 1) the numerator denotes the connectivity of two miRNA-related disease sets defined by the sum of the path weights between them; and 2) the denominator is used for suppressing miRNA related with many diseases. In addition, MFSP has the symmetric property as the miRNA-miRNA path matrix *P* is a symmetric matrix, which makes it useful in many applications^50^.

The symmetric property shows that MFSP has the more general symmetric property for different maximum transferring times. This property is useful for many applications. For instance, if the functional similarity of miRNA pair can be measured and the similarity is symmetric, MIDP algorithm^9^ can be applied in the similarity matrix directly. Moreover, Cluster One^51^ method can be performed on the miRNA functional network, a weighted undirected graph which is constructed based on the functional similarity of miRNA pair.

Assume that a directed acyclic graph and miRNA-disease associations are shown in Fig. 5, we simply showed the procedure for calculating the MFSP between *m*_1_ and *m*_2_. The maximum transferring times *b* is set as 2 and the weight ratio *a* is set as 0.6. We can obtain the feature vector *V*_*d*1_, *V*_*d*2_, *V*_*d*3_, *V*_*d*4_, *V*_*d*5_ and *V*_*d*6_ to calculate the disease semantic similarity matrix *S*_*DD*_ using cosine similarity (shown in Fig. 5). Then, transferring matrixes for different transferring times are achieved by formula (3) as follows:


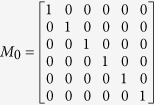



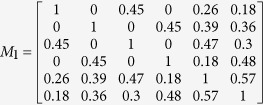



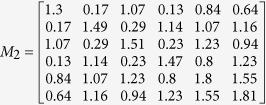


We can obtain the adjacency matrix *R*_*MD*_ of miRNA-disease based on miRNA-disease association is:


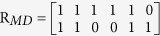


After combined the different transferring matrix and the adjacency matrix of miRNA-disease, the miRNA-miRNA path matrix *P* is:


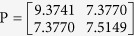


As a result, the functional similarity of *m*_1_ and *m*_2_ is calculated to be (2*7.377)/(9.3741 + 7.5149) = 0.8736, and the functional similarity of *m*_1_ − *m*_1_ pair is (2*9.3741)/(9.3741 + 9.3741) = 1.

MFSP is implemented in C++ and can be downloaded at https://github.com/KDDing/MFSP.

## Additional Information

**How to cite this article**: Ding, P. *et al*. A path-based measurement for human miRNA functional similarities using miRNA-disease associations. *Sci. Rep.* **6**, 32533; doi: 10.1038/srep32533 (2016).

## Supplementary Material

Supplementary Information

Supplementary Material 1

Supplementary Material 2

## Figures and Tables

**Table 1:** P-values obtained by Wilcoxon rank-sum testing and Kruskal-Wallis testing functional similarity of the intra-family, inter-family and randomly selected miRNAs.

	Wilcoxon rank-sum test			Kruskal-Wallis test
	intra-inter	intra-random	inter-random	Intra-inter-random
Wang’s method	0.00E-00	0.00E-00	1.30E-03	0.00E-00
Xuan’s method	0.00E-00	0.00E-00	1.10E-03	0.00E-00
CSD+BMA	0.00E-00	0.00E-00	9.02E-04	0.00E-00
MFSP	0.00E-00	0.00E-00	1.94E-04	0.00E-00

**Table 2:** P-values obtained by Wilcoxon rank-sum testing and Kruskal-Wallis testing functional similarity of the intra-cluster, inter-cluster and ran-domly selected miRNAs.

	Wilcoxon rank-sum test			Kruskal-Wallis test
	intra-inter	intra-random	inter-random	Intra-inter-random
Wang’s method	3.02E-211	3.23E-206	1.53E-02	1.17E-209
Xuan’s method	5.58E-223	1.15E-217	1.27E-02	2.23E-221
CSD+BMA	4.46E-226	1.09E-220	1.20E-02	1.79E-224
MFSP	0.00E-00	0.00E-00	2.50E-03	0.00E-00

**Table 3:** AUC of MIDP applied on miRNA functional similarity network constructed by different methods.

Disease	MFSP	Wang’s method	Xuan’s method
Melanoma	**0.948**	0.9467	0.9469
Hepatocellular Carcinoma	0.9674	**0.9678**	0.9671
Breast Neoplasms	**0.9592**	0.9589	0.9588
Colorectal Neoplasms	**0.9608**	0.9583	0.9578
Stomach Neoplasms	**0.9435**	0.9414	0.9402
Average AUC	**0.95578**	0.95462	0.95416
