# Supplementary material for: A path-based measurement for human miRNA functional similarities using miRNA-disease associations
Source: Sci Rep. 2016 Sep 2;6:32533. doi: 10.1038/srep32533 (PMC5009308; doi:10.1038/srep32533)
Supplement: Supplementary Information [file srep32533-s1.pdf]

A path-based measurement for human miRNA functional similarities using miRNA-disease associations

Pingjian Ding[1], Jiawei Luo[1,*], Qiu Xiao[1], and Xiangtao Chen[1]
[1]College of Computer Science and Electronic Engineering, Hunan University, Changsha, 410083, China

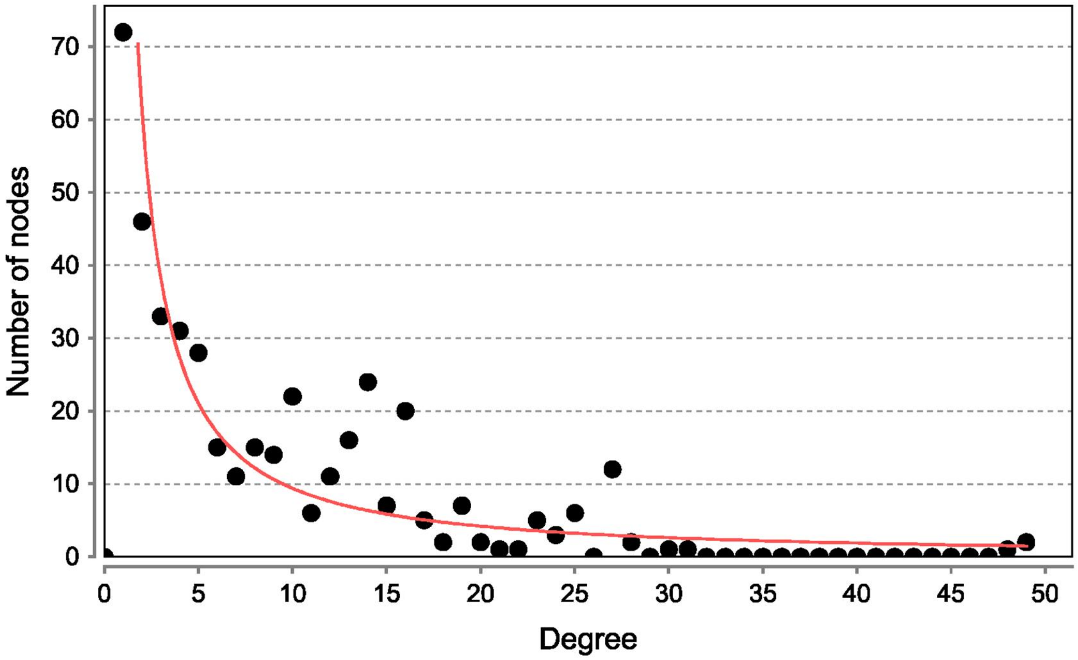

Supplementary Figure 1. Degree distribution of the miRNA functional network

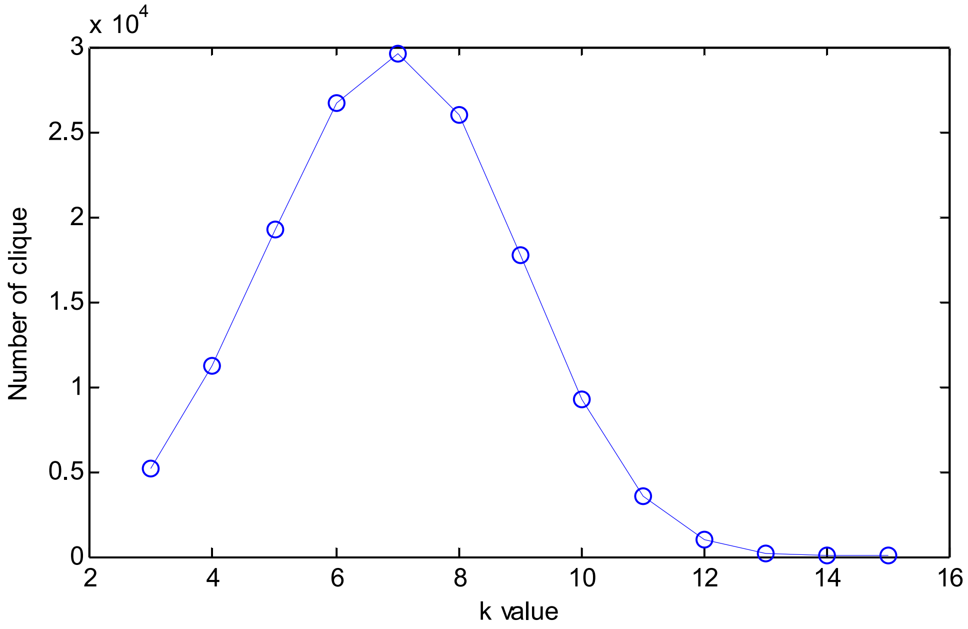

Supplementary Figure 2. Number of cliques at different k-values

Supplementary Table 1. P-values with different maximum transferring times *b*

	family				cluster			
	Wilcoxon rank-sum			Kruskal-Wallis	Wilcoxon rank-sum			Kruskal-Wallis
b	intra-inter	intra-random	inter-random	intra-inter-random	intra-inter	intra-random	inter-random	intra-inter-random
0	0.00E-00	0.00E-00	3.22E-04	0.00E-00	4.75E-251	1.16E-244	0.0081	2.01E-249
1	0.00E-00	0.00E-00	6.42E-04	0.00E-00	1.52E-241	8.69E-236	0.0094	6.31E-240
2	0.00E-00	0.00E-00	3.89E-04	0.00E-00	3.32E-279	1.51E-272	0.0052	1.48E-277
3	0.00E-00	0.00E-00	2.63E-04	0.00E-00	6.99E-307	1.47E-299	0.0034	3.28E-305
4	0.00E-00	0.00E-00	2.15E-04	0.00E-00	0.00E+00	0.00E+00	0.0027	3.3776e-319
5	0.00E-00	0.00E-00	1.94E-04	0.00E-00	0.00E+00	0.00E+00	0.0025	0.00E+00
6	0.00E-00	0.00E-00	1.83E-04	0.00E-00	0.00E+00	0.00E+00	0.0023	0.00E+00
7	0.00E-00	0.00E-00	1.77E-04	0.00E-00	0.00E+00	0.00E+00	0.0022	0.00E+00
8	0.00E-00	0.00E-00	1.74E-04	0.00E-00	0.00E+00	0.00E+00	0.0022	0.00E+00
9	0.00E-00	0.00E-00	1.72E-04	0.00E-00	0.00E+00	0.00E+00	0.0022	0.00E+00
10	0.00E-00	0.00E-00	1.70E-04	0.00E-00	0.00E+00	0.00E+00	0.0022	0.00E+00

Supplementary Table 2. P-values with different weight ratio *a*

	family				cluster			
	Wilcoxon rank-sum			Kruskal-Wallis	Wilcoxon rank-sum			Kruskal-Wallis
a	intra-inter	intra-random	inter-random	intra-inter-random	intra-inter	intra-random	Inter-random	intra-inter-random
0.1	0.00E-00	0.00E-00	5.03E-04	0.00E-00	1.01E-247	8.07E-242	8.60E-03	4.26E-246
0.2	0.00E-00	0.00E-00	4.80E-04	0.00E-00	2.59E-253	2.81E-247	7.80E-03	1.10E-251
0.3	0.00E-00	0.00E-00	4.12E-04	0.00E-00	1.42E-266	3.20E-260	6.40E-03	6.18E-265
0.4	0.00E-00	0.00E-00	3.18E-04	0.00E-00	1.27E-287	9.22E-281	4.60E-03	5.78E-286
0.5	0.00E-00	0.00E-00	2.40E-04	0.00E-00	0.00E-00	1.00E-302	3.20E-03	0.00E-00
0.6	0.00E-00	0.00E-00	1.94E-04	0.00E-00	0.00E-00	0.00E-00	2.50E-03	0.00E-00
0.7	0.00E-00	0.00E-00	1.71E-04	0.00E-00	0.00E-00	0.00E-00	2.00E-03	0.00E-00
0.8	0.00E-00	0.00E-00	1.61E-04	0.00E-00	0.00E-00	0.00E-00	1.80E-03	0.00E-00
0.9	0.00E-00	0.00E-00	1.58E-04	0.00E-00	0.00E-00	0.00E-00	1.70E-03	0.00E-00
1.0	0.00E-00	0.00E-00	1.58E-04	0.00E-00	0.00E-00	0.00E-00	1.60E-03	0.00E-00
